# An Unusual Position of Retromandibular Vein in Relation to Facial Nerve: A Rare Case Report

**DOI:** 10.1155/2018/3919762

**Published:** 2018-11-06

**Authors:** Nabin Lageju, Prabhat Chandra Thakur, Pravin Kumar Jaiswal

**Affiliations:** ^1^Department of ENT-HNS, Nepal Police Hospital, Kathmandu, Nepal; ^2^Department of Head and Neck Oncology, Nepal Cancer Hospital, Lalitpur, Nepal

## Abstract

Knowledge of different anatomical structures is very important in parotid surgery to preserve facial nerve. Retromandibular vein is one of the landmarks used to identify facial nerve. So, the relation of the vein with facial nerve is very important in parotid surgery. The typical position of RMV is deep to facial nerve in almost 88% of cases reported in various literatures. Here, we present an unusual position of RMV found during parotid surgery for pleomorphic adenoma.

## 1. Introduction

The retromandibular vein (RMV) is formed by the union of superficial temporal vein and maxillary vein and descends in the substance of the parotid gland. RMV is closely related with facial nerve within the parotid tissue. Identification and preservation of facial nerve is one of the crucial steps in the parotid surgery [1, 2], and RMV is one of the landmarks to identify facial nerve in parotid surgery [3]. Complications such as bleeding and damage to facial nerve may occur if the surgeon is not aware about the different anatomical variations of RMV in relation to facial nerve [4]. This study presents one of the rarest variations of relationship between RMV and facial nerve found during parotid surgery.

## 2. Case Report

**Figure 1:**
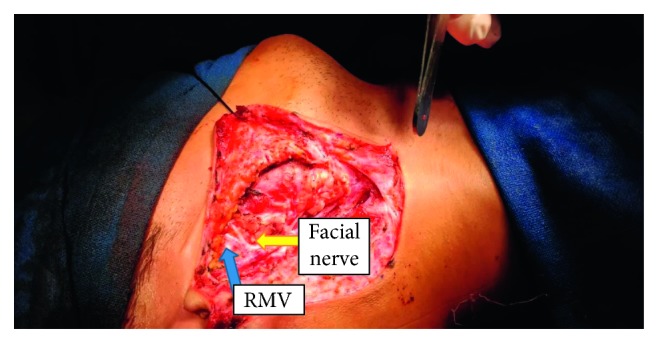
Preoperative view after adequate parotidectomy showing RMV lateral to facial nerve branches.

A 24-year-old male patient presented with gradually progressive swelling in the right side of the cheek and below the earlobe for last one year with no complaint of pain, fever, redness over the skin, or any weakness of facial musculature. On physical examination, a 3 × 2 cm firm, nontender, and mobile swelling was present in the right parotid region. Ultrasound examination showed a 2.7 × 1.5 cm well-defined swelling in the superfacial lobe of the right parotid gland with minimal vascularity. A fine-needle aspiration cytology revealed pleomorphic adenoma of the right parotid gland. With this diagnosis, right adequate parotidectomy was planned. During surgery after identification of facial nerve, while tracing branches of facial nerve forwards RMV was found to be crossing the two main trunks of facial nerve remaining lateral as shown in Figure 1. All the branches of facial nerve were identified, and adequate parotidectomy was done.

## 3. Discussion

Facial nerve is one of the most important structures to be preserved in parotid surgery [1] as it controls the muscles of facial expression. During parotid surgery, there are important landmarks such as tragal pointer, posterior belly of digastric, stylomastoid foramen, RMV, and tympanomastoid suture line to identify facial nerve [5, 6].

Localization of RMV helps in identification and preservation of facial nerve and its branches. In most of the cases (88%), RMV is medial to facial nerve, but in some cases (11.83%), anatomical variations of RMV were found [7] and this creates risk of bleeding and damage to facial nerve [4, 6]. RMV may be found lateral to the lower division and medial to the upper division and may also be detected lying anterior to the facial nerve as a ring around the nerve branches or as a fork formed by the vein branches [1, 6, 8].

A cadaveric study was conducted to examine the correlation of an anatomy of the marginal mandibular branch of the facial nerve and its surrounding tissues [9]. This study investigated 85 facial halves and found that 83% of the specimens had inferior division of the facial nerve crossed laterally to the RMV, whereas 17% had inferior division of the facial nerve crossed medially to the RMV [9]. The study done by Kopuz et al. reported that, in 15% of the cases, the variations involved both sides [10].

## 4. Conclusion

An anatomical variation was found in relation between facial nerve and RMV, and this might increase the risk of bleeding and facial nerve injury during parotid surgery. Therefore, surgeons must be aware of this important fact.
